# Arbeitsorganisatorische Strategien und Maßnahmen zum Pandemiemanagement in der (akut-)stationären Pflege: Ergebnisse von Szenario-Workshops

**DOI:** 10.1055/a-2764-6645

**Published:** 2026-03-02

**Authors:** Maria Zink, Niels Jansen, Florence Zurfluh, Dodo Vögler, Marlen Melzer

**Affiliations:** 1 Arbeitsgestaltung bei personenbezogenen Dienstleistungen (3.3), Bundesanstalt für Arbeitsschutz und Arbeitsmedizin Standort Dresden, Dresden, Germany; 2 Institut für Sozialmedizin, Arbeitsmedizin und Public Health, Universität Leipzig Medizinische Fakultät, Leipzig, Germany; 3 Ellery Studio, Berlin, Germany

**Keywords:** Krisenmanagement, beruflich Pflegende, Zukunftsforschung, transdisziplinär, Pflegepolitik, crisis management, nurses, foresight methods, transdisciplinary approach, nursing policy

## Abstract

**Ziel:**

Ziel des Szenario-Workshops war es, in einem trans- und interdisziplinären Diskurs geeignete Maßnahmen und Strategien zu entwickeln, die das Pandemiemanagement in (akut-)stationären Pflegeeinrichtungen verbessern und somit zur Entlastung beruflich Pflegender beitragen können.

**Methodik:**

Es wurden zwei Szenario-Workshops durchgeführt, einer für den Bereich der Akutpflege und einer für den der Langzeitpflege. Die Workshops folgten einem kollaborativen, transdisziplinären Ansatz, der Perspektiven aus der Pflegepraxis, -forschung und relevanten Institutionen integrierte. Mithilfe von Methoden der Zukunftsforschung wie „Futures Wheel“ oder „Ideation Canvas“ wurden potenzielle Ansatzpunkte zur Optimierung des Pandemiemanagements entwickelt. An den zweitägigen Workshops nahmen insgesamt 38 Teilnehmende teil (Akutpflege: 18; Langzeitpflege: 20)

**Ergebnisse:**

Die Teilnehmenden erarbeiteten Ergebnisse auf struktureller und arbeitsorganisatorischer Ebene sowie kurz- und langfristig wirksame Maßnahmen und Strategien. Zentrale Erkenntnisse betrafen Themenfelder wie Kommunikation, interne Organisation, Schnittstellenmanagement und der Einbezug von Pflegepraktiker:innen. Beispiele sind die (politische) Stärkung der Pflege und deren Partizipation, der Aufbau digitaler und interdisziplinärer Strukturen sowie von Skill-Grade-Mix-Teams. In beiden Workshops wurden eine verbindliche pflegerische Interessensvertretung und klare Kompetenzregelungen für den Pflegeberuf gefordert.

**Schlussfolgerung:**

Effektives Krisenmanagement in stationären Pflegeeinrichtungen erfordert einen systemischen Ansatz, der Maßnahmen auf allen Ebenen (Mikro, Meso, Makro) integriert. Ein koordiniertes Zusammenspiel von Politik, Wissenschaft und Praxis ist entscheidend, um die Resilienz des Systems zu stärken und die Arbeitsbedingungen in den Gesundheitseinrichtungen langfristig zu verbessern.

## Abstract (en)

**Objective:**

The aim of this study was to develop suitable measures and strategies through a trans- and interdisciplinary discourse to improve pandemic management in (acute) inpatient care facilities (clinics, nursing homes) and thereby reduce the burden on nursing staff.

**Methods:**

Two scenario-based workshops were conducted—one focused on acute care and the other on long-term care. Following a collaborative, transdisciplinary approach, the workshops integrated perspectives from nursing practice, research, and relevant institutions. Using foresight methods such as the Futures Wheel and Ideation Canvas, participants co-developed potential solutions for optimizing pandemic preparedness and response. A total of 38 participants took part in the two-day workshops (acute care: 18; long-term care: 20).

**Results:**

Participants developed outcomes at both structural and organizational levels, identifying short- and long-term effective strategies and interventions. Key findings addressed areas such as communication, internal organization, interface management, and the involvement of nursing practitioners. Examples include the (political) empowerment and increased participation of nurses, the development of digital and interdisciplinary structures, including skill-grade mix teams. Across both care settings, participants emphasized the need for formal nursing representation and clearly defined professional roles and responsibilities.

**Conclusion:**

Effective crisis management in inpatient care settings requires a systemic approach that integrates actions at the micro, meso, and macro levels. Coordinated collaboration between policy, science, and practice is essential to strengthen system resilience and sustainably improve working conditions in healthcare.

## Einleitung

Die COVID-19-Pandemie hat die bereits bestehenden strukturellen Anforderungen in (akut-)stationären Pflegeeinrichtungen drastisch verschärft. Professionell Pflegende sahen sich einer Vielzahl an Stressoren ausgesetzt. Die psychischen und organisatorischen Herausforderungen legten dabei systemische Schwachstellen offen und verdeutlichten die Dringlichkeit eines vorausschauenden, resilienten Krisenmanagements in der Pflege.

### Pandemiemanagement und Herausforderungen für das Pflegepersonal

Obwohl Expert:innen, die in der Risikoanalyse des Deutschen Bundestags 1 bereits vor einem pandemischen Virusausbruch gewarnt hatten, stellte die COVID-19-Pandemie, besonders zu Beginn, das Krisenmanagement von (akut-)stationären Einrichtungen vor erhebliche Herausforderungen. Gründe dafür waren unter anderem Mangel an Schutzausrüstung und Personal 2 3 4 . In Deutschland prägten föderale Strukturen und gesetzliche Vorgaben das Pandemiemanagement (siehe Abb. 1 ). Daraus entstanden Diskrepanzen zwischen einheitlichen Regelungen und lokalem Handlungsbedarf. Träger stationärer Einrichtungen mussten ständig wechselnde Maßnahmen umsetzen – oft ohne ausreichende Vorbereitung 5 6 .

**Abb. 1:**
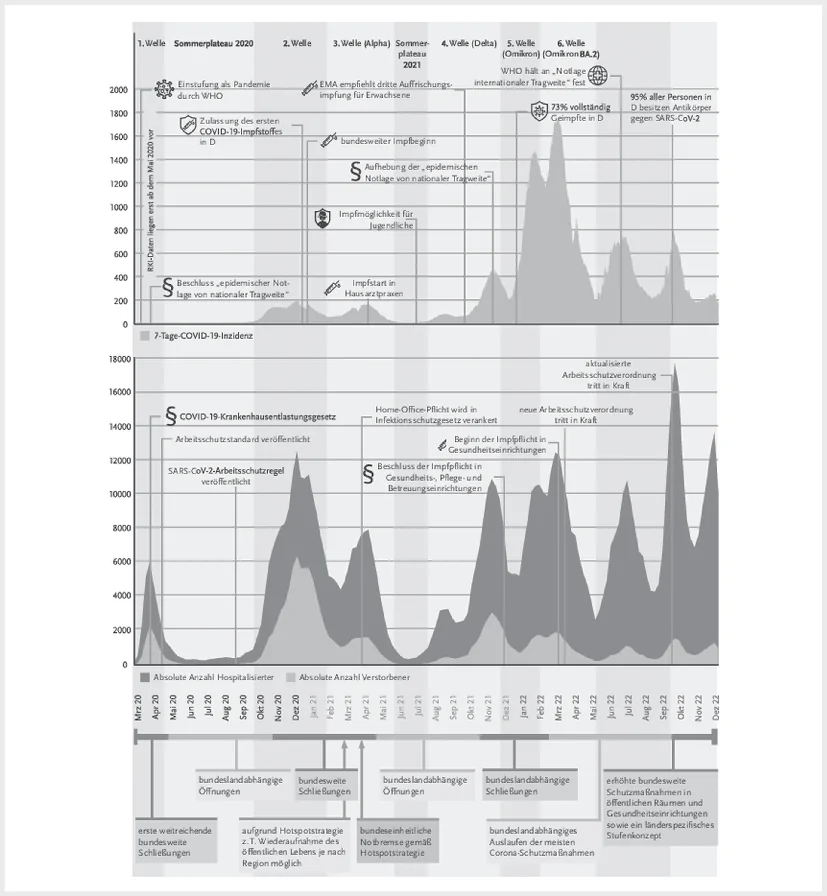
Darstellung des Pandemieverlaufes in Deutschland. Oben: 7 Tage COVID-19 Inzidenz und gesamtgesellschaftlich relevante Ereignisse; Mitte: absolute Anzahl Hospitalisierter und Verstorbener sowie arbeitsrelevante Ereignisse; unten: Phasen der gesetzlich vorgegebenen Schließungen und Öffnungen 7 . Quelle: Bundesanstalt für Arbeitsschutz und Arbeitsmedizin, Dortmund.

Der Pandemieverlauf sowie das nationale, regionale und einrichtungsinterne Pandemiemanagement führten zu erheblichen Herausforderungen im gesamten Arbeitssystem der Pflegenden (z. B. in der Interaktionsarbeit mit Pflegebedürftigen und Angehörigen, innerhalb des Teams 6 ). Besonders betroffen waren gesundheitsrelevante Arbeitsmerkmale wie eine erhöhte Arbeitsintensität 6 , erschwerte Kommunikation 7 , mehr Verantwortung für Pflegebedürftige 8 9 10 , und Veränderungen der Arbeitsorganisation 5 11 . Auch die soziale Unterstützung durch das Team, eine zentrale Ressource für Pflegende, wurde durch die Vorgaben des Social Distancing eingeschränkt 6 . Zudem führte das erhöhte Infektionsrisiko in Kombination mit Quarantäneregelungen zu einer steigenden Anzahl von Arbeitsunfähigkeitsmeldungen und einer weiterhin hohen Krankheitsrate im Jahr 2023 12 13 .

### Lehren der Pandemie für das Krisenmanagement

Die COVID-19-Pandemie hat gezeigt, dass Pflegeeinrichtungen strukturell und organisatorisch widerstandsfähiger werden müssen, um künftige Krisen bewältigen zu können. Arbeitsorganisation spielt dabei im operativen Krisenmanagement eine zentrale Rolle und ist ein integraler Bestandteil der Gestaltung von Arbeitssystemen 14 . Angesichts wachsender Risiken, z. B. aufgrund der Klimakrise, liefern die dargestellten Workshops praxisnahe Erkenntnisse zur Optimierung des Krisenmanagements in (akut-)stationären Einrichtungen. Der Erhalt der physischen und psychischen Gesundheit von Pflegenden ist entscheidend, um die Versorgung trotz Personalengpässen langfristig sicherzustellen. Zukunftsorientiertes Krisenmanagement muss über akute Krisenpläne hinausgehen – nicht zuletzt, weil es erforderlich sein wird, auf unterschiedliche Krisenszenarien (z. B. Extremwetter, Cyberangriffe, 15 ) vorbereitet zu sein. Methoden der Zukunftsforschung können dabei unterstützen, tragfähige Maßnahmen und Strategien zu entwickeln.

Ziel dieser Studie war es, im Rahmen eines trans- und interdisziplinären Diskurses wirkungsvolle Maßnahmen und Strategien zu erarbeiten, die das Pandemiemanagement in (akut-)stationären Pflegeeinrichtungen optimieren und dadurch die Arbeitsbelastung von Pflegenden nachhaltig reduzieren können. Es sollte folgende Frage beantwortet werden:

Welche arbeitsorganisatorischen Maßnahmen und Strategien können das Pandemiemanagement (akut-)stationärer Einrichtungen zukünftig stärken und zur Entlastung beruflich Pflegender beitragen?

## Methode

Es wurden separate Szenario-Workshops für die stationäre Akut- und Langzeitpflege durchgeführt, um den spezifischen Herausforderungen in beiden Settings während der Pandemie gerecht zu werden.

### Zukunftsszenarien als Instrument zur Handlungsorientierung

Zukunftsforschung dient dazu, unter Bedingungen von Unsicherheit Orientierung für gegenwärtiges Handeln zu schaffen. Statt einzelner Prognosen werden verschiedene mögliche, wahrscheinliche und wünschenswerte Zukünfte analysiert, um Zielbilder zu entwickeln und geeignete Maßnahmen zum Erreichen dieser abzuleiten. Szenarien-Arbeit ist ein zentrales Werkzeug der Zukunftsforschung: Sie strukturiert komplexe Zusammenhänge, zeigt Entwicklungspfade auf, eröffnet Handlungsspielräume und unterstützt strategisches Handeln.

### Methodisches Vorgehen

Die Vorgehensweise der Workshops folgte einem transdisziplinären Ansatz, der Theorie- und Praxiswissen miteinander verzahnt. Durch die Integration heterogener Perspektiven sollte ein anwendungsorientiertes Verständnis geschaffen und realisierbare Maßnahmen entwickelt werden. Zukunftsforschung wird hier als gestaltender Prozess verstanden, der durch kollaborative Szenarien-Arbeit praxisnahe, strategisch anschlussfähige Lösungen hervorbringt.

Die Workshops fokussierten auf ein offenes Situationsszenario, strebten die Entwicklung konkreter Handlungsempfehlungen an und orientierten sich an der Methode der „Zukunftswerkstatt“ nach Jungk und Müllert 16 . Ergänzend wurden Instrumente aus der Zukunftsforschung (z. B. Futures Wheel) und dem Design Thinking (z. B. Ideation Canvas) eingesetzt.

Die Workshops gliederten sich in drei Phasen:

1. *Analysephase:* In moderierten Gruppen wurden persönliche Erfahrungen aus der COVID-19-Pandemie gesammelt und mithilfe der Problembaum-Methode strukturiert.
2. *Zukunftsphase:* Aufbauend auf „Was-wäre-wenn“-Fragen erarbeiteten die Teilnehmenden in Futures Wheels mögliche Szenarien und deren direkte wie indirekte Auswirkungen auf Mikro-, Meso- und Makroebene.
3. *Praxisphase:* Die Ergebnisse der vorherigen Phasen wurden in konkreten Maßnahmen verdichtet. Mittels Aufwand-Einfluss-Matrix und Ideation Canvas entwickelten die Gruppen umsetzungsorientierte Vorschläge zur Verbesserung der Arbeitsorganisation in zukünftigen Krisensituationen.

### Akquisition Teilnehmender

Die Teilnehmenden-Auswahl folgte einem trans- und interdisziplinären Ansatz. Dabei wurden sowohl Fachpersonen aus der Pflegepraxis und -forschung als auch Vertreter:innen relevanter Institutionen berücksichtigt. Ziel war es, sowohl horizontale als auch vertikale Schnittstellen des Gesundheitswesens abzubilden. Eine Stakeholder-Analyse ermöglichte die gezielte Auswahl von insgesamt 38 Teilnehmenden aus der Akut- und Langzeitpflege (18 bzw. 20 Personen).

### Auswertung

Die Workshop-Ergebnisse wurden fotografisch festgehalten. Zusätzlich fertigte das Forschungsteam Protokolle und Notizen an. Diese Materialien wurden zunächst von mindestens zwei Personen zusammengefasst und anschließend in mehreren Sitzungen im sechsköpfigen Team diskutiert.

## Ergebnisse

### Analysephase: Herausforderungen während der Pandemie

Setting-übergreifende und -spezifische Herausforderungen wurden von den Teilnehmenden genannt. Eine vertiefende Analyse ergab vier zentrale Problemfelder: Psychische Belastung und inflexibles Krisenmanagement, physische und psychische Überlastung des Pflegepersonals, mangelnde interne und externe Partizipation und fehlende Entscheidungsspielräume von Pflegepersonen, fehlende Orientierung und unsichere Kommunikation (siehe Tab. 1 ).

**Table:** **Tab. 1** Herausforderungen und Problemfelder struktureller und organisationaler Defizite während der COVID-19 Pandemie.

Herausforderungen beider Pflegesettings	Herausforderungen in der Langzeitpflege
Ressourcenmangel (etwa bei Schutzkleidung oder Personal) hohe psychische Belastungen durch Unsicherheit, Überlastung und häufige Regeländerungen Spannungsfeld zwischen Eigeninitiative und Fremdbestimmung unzureichende Kommunikation mangelnde gesellschaftliche Wertschätzung Ambivalenz in der digitalen Umstellung Diskrepanz zwischen professionellem Selbstverständnis und dem Gefühl, in der Krise allein gelassen worden zu sein	Isolation der Bewohnenden „stille Triage“-Erleben „einsame Sterben“ Erster Vertrauensbruch durch das Gefühl des gesellschaftlichen Ausschlusses, das durch mediale Kritik zusätzlich verstärkt wurde Zweiter Vertrauensbruch durch erlebte Abwertung nach der Pandemie – etwa durch misstrauisches Verhalten externer Akteure Spannungsfelder: Arbeitsethos vs. Instrumentalisierung, solidarisches Miteinander im Team vs. gesellschaftlicher Rückzug, Informationsflut und fehlender Orientierung
**Herausforderungen in der Akutpflege**	
häufiger Kontakt mit schwerem Krankheitsverlauf und Tod (besonders belastend für jüngere Kolleg:innen)	ethische Fragen zu Behandlungsabbruch und Priorisierung Variation der psychischen Belastung in Abhängigkeit der Intensität der Akutsituationen
**Vier zentrale Problemfelder**	
*Physische und psychische Überlastung des Pflegepersonals***Ursachen** : u. a. Personalmangel, fehlende bauliche und digitale Infrastruktur, unklare Notfallkonzepte **Folgen** : Belastungen von sozialem Rückzug bis zu posttraumatischen Symptomen	*Mangelnde interne und externe Partizipation und fehlende Entscheidungsspielräume von Pflegepersonen***Ursachen** : ausgeprägte Top-down-Kultur, fehlende Innovationsfreiräume, Fehlen einer bundesweiten Berufsvertretung **Verbesserung** : mehr Handlungskompetenz und systemischer Wertschätzung
*Psychische Belastung und inflexibles Krisenmanagement***Ursachen** : Vermittlung unklarer Regelungen, fehlende Unterstützungsangebote, Verlust an Lebensqualität für Bewohnende und rigide Schutzmaßnahmen in der Langzeitpflege **Diskussion** : Sinnhaftigkeit einheitlicher Regelungen **Verbesserung** : kontextbezogene, gemeinsam entwickelte Maßnahmen	

### Zukunftsphase: Szenarien-Entwicklung und systemisches Denken

In der Zukunftsphase entwickelten die Teilnehmenden mithilfe von mehr als 90 erarbeiteten „Was-wäre-wenn“-Fragen und der Futures-Wheel-Methode vielfältige Szenarien zu Themen wie Digitalisierung, Arbeitsorganisation und Formen der Krisensteuerung (siehe Abb. 2 ).

**Abb. 2:**
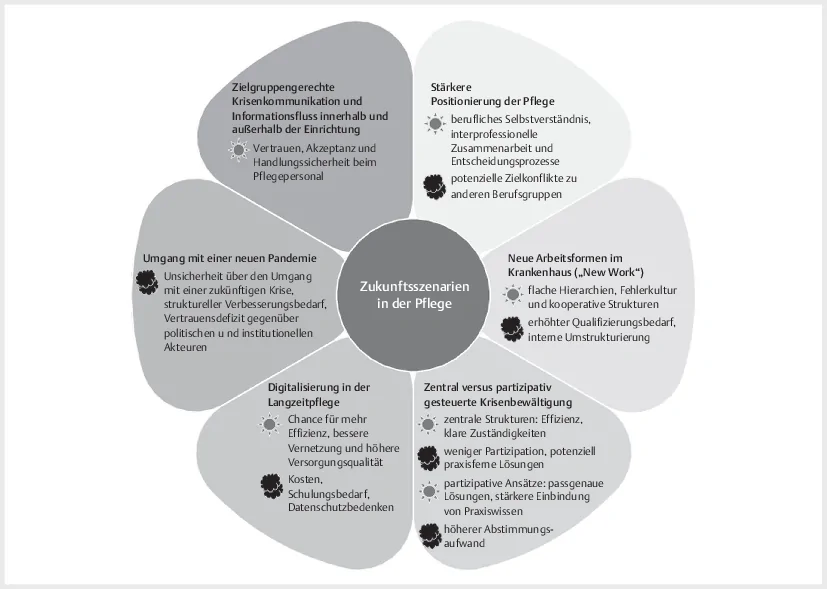
Sechs Zukunftsszenarien für den Bereich der Pflege. Quelle: Bundesanstalt für Arbeitsschutz und Arbeitsmedizin, Dortmund.

### Praxisphase: Von Ideen zu umsetzbaren Maßnahmen

Die zuvor erarbeiteten Maßnahmen wurden in einer Aufwand-Einfluss-Matrix priorisiert und im Ideation Canvas weiter ausgearbeitet.

### Maßnahmen in der Akutpflege

#### Strukturelle Maßnahmen

Im Fokus standen die Stärkung der Pflege als Beruf und gesellschaftliche Instanz, etwa durch Imagekampagnen oder einer stärkeren Vertretung gegenüber Politik und Öffentlichkeit. Benannte Ziele dessen sind eine bessere Sichtbarkeit pflegerischer Belange sowie eine Aufwertung des Berufsbildes.

Langfristige und strategisch relevante Vorschläge beinhalteten verbindliche Arbeitsschutzregelungen **,** insbesondere zum Erhalt und zur Förderung psychischer Gesundheit **,** New-Work-orientierte Arbeitszeitmodelle **,** verstärkte pflegewissenschaftliche Forschung sowie die Einrichtung einer Pflegekammer als Interessenvertretung des Berufsstandes.

#### Arbeitsorganisatorische Maßnahmen

Die Teilnehmenden legten einen Fokus auf die Umsetzung flexibler Arbeitszeitmodelle, psychosoziale Unterstützungsangebote sowie inter- und intradisziplinäre Ausbildungsformate. Zudem betonten die Teilnehmenden die Notwendigkeit sektorübergreifender Zusammenarbeit – insbesondere zwischen Rettungsdiensten, Kliniken und Pflegeeinrichtungen – zur Verbesserung des Informationsflusses und der Versorgungsqualität. Die Förderung einer reflektierenden Teamkultur sowie Mentoring-Angebote wurde ebenfalls als zentrale Maßnahmen zur Stärkung des Wohlbefindens im Arbeitsalltag während einer Pandemie hervorgehoben.

Kurzfristig wirksame arbeitsorganisatorische Maßnahmen mit hohem Einfluss sind nach Ansicht der Teilnehmenden klare Aufgabenbeschreibungen für Pflegepersonal, informelle Austauschformate sowie interdisziplinäre Ausbildungsangebote.

### Maßnahmen in der Langzeitpflege

#### Strukturelle Maßnahmen

Zentrale Vorschläge umfassten die Gründung lokaler Krisenpräventionsbündnisse, ein zentralisiertes digitales Meldesystem für pandemierelevante Daten sowie die Stärkung der im SGB XI verankerten, nicht-profitorientierten Ausrichtung der Pflege. Auch ein Modell zur Digitalisierung, inklusive Anbindung an die Telematikinfrastruktur, das einheitlichen, einrichtungsübergreifenden Standards folgt, wurde angeregt, um während einer Pandemie Prozesse zu optimieren. Darüber hinaus schlugen die Teilnehmenden als Maßnahme zur besseren Vernetzung und sozialen Integration die Öffnung von Pflegeeinrichtungen in den Sozialraum vor.

Langfristige und strategisch relevante Vorschläge beinhalteten das Definieren von Eskalationsstufen mit Leitlinien für Krisen, regionale Netzwerkbildung zur Ressourcenbündelung sowie die digitale Transformation in Bereichen wie Kommunikation und Koordination. Auch eine Stärkung der berufsständischen Vertretung wurde als hilfreich erachtet.

#### Arbeitsorganisatorische Maßnahmen

Hier lag der Schwerpunkt auf Schulungen für Mitarbeitende, Multiplikator:innen und Führungspersonen im Umgang mit Krisensituationen. Der Aufbau klarer Kommunikations- und Netzwerkstrukturen – sowohl innerhalb als auch zwischen Einrichtungen – wurde als zentral angesehen, um im Krisenfall schnelle und koordinierte Reaktionen zu ermöglichen.

Kurzfristig wirksame arbeitsorganisatorische Maßnahmen mit hohem Einfluss sind nach Ansicht der Teilnehmenden der Ausbau von Telematikinfrastruktur und interner Digitalisierung, wobei auf die Notwendigkeit zur zielgerichteten Kommunikation aktueller Vorgaben gegenüber Mitarbeitenden hingewiesen wurde, um Akzeptanz der Technik zu fördern.

#### Schwerpunkte in beiden Settings

Beide Workshops forderten die Einrichtung einer berufsständischen Vertretung, klare Kompetenzzuweisungen der Pflegenden und dauerhafte Krisenstäbe mit Pflegebeteiligung. Übergreifend wurde die Profitorientierung im Gesundheitswesen kritisch hinterfragt – als strukturelles Hindernis für resiliente, an den Bedürfnissen von Pflegenden und Patient:innen orientierte Organisationen. Dies basierte auf der Erfahrung, dass die Einsparungsmaßnahmen der vergangenen Jahre – etwa die Reduktion von Pflegepersonal, Bettenkapazitäten und Vorratshaltung sowie die Unterfinanzierung des öffentlichen Gesundheitsdienstes – sich während der COVID-19-Pandemie negativ auf die Versorgung und die Gesundheit des Personals auswirkten.

## Diskussion

Die Ergebnisse der Szenario-Workshops verdeutlichen den dringenden Bedarf, Pflegeeinrichtungen strukturell und organisatorisch krisenfester zu gestalten. Dabei reicht es nicht aus, allein Maßnahmen auf der Mikroebene – etwa bei den Teams oder einzelnen Pflegenden – zu etablieren. Vielmehr bedarf es eines integrierten Ansatzes, der aufeinander abgestimmte Veränderungen auf allen Ebenen (Mikro-, Meso- und Makro-Ebene) umfasst. Dabei stehen vor allem Themenfelder wie Kommunikation, interne Organisation, Schnittstellenmanagement und die Partizipation von Pflegepraktiker:innen im Fokus. Ähnliche Aspekte wurden unteranderem auch in Studien vor der Pandemie, insbesondere zu Problemen im Schnittstellenmanagement 17 , sowie in Untersuchungen zum verbesserten Pandemiemanagement in Gesundheitsämtern (Kommunikation, interne Organisation, Partizipation, 18 ) identifiziert. Dies deutet darauf hin, dass diese Themen aus Sicht von Akteur:innen im Gesundheitswesen allgemein von hoher Relevanz sind.

Zentrale Ansatzpunkte struktureller und organisatorischer Optimierungen liegen in der Einrichtung multiprofessioneller Krisenstäbe, der regelmäßigen Überarbeitung und dem Üben von Krisenplänen sowie in der Flexibilisierung von Arbeitsorganisation und Arbeitszeitmodellen. Ein besonderer Fokus sollte auf der Stärkung der Teamkultur, psychosozialer Unterstützung und Supervisionen sowie der institutionalisierten Fortbildung des Pflegepersonals und deren Führungspersonen in Bereichen wie psychische Gesundheit, Digitalisierung und ethische Entscheidungsfindung liegen.

Darüber hinaus wurde der Aufbau von Skill-Grade-Mix-Teams als strategisch sinnvoll erachtet, um während Krisenzeiten vorhandene Kompetenzen gezielter im Pflegealltag zu nutzen. Eine professionelle Krisenkommunikation beeinflusst die Gesundheit der Pflegenden 19 . Diese muss – intern wie extern – frühzeitig vorbereitet und regelmäßig evaluiert werden.

Die Digitalisierung wurde als Schlüsselkomponente für zukunftsfähige Pflegeeinrichtungen identifiziert. Dabei ist ein partizipativer Einführungsprozess entscheidend, um Akzeptanz zu fördern und Technologien passgenau zu implementieren (vgl. 20 ).

Auf Meso-Ebene gilt es, Netzwerke und regionale Kooperationen auszubauen, um Ressourcen zu bündeln, Erfahrungen auszutauschen und gemeinsam auf Krisen reagieren zu können (vgl. 11 ). Modelle wie der Einbezug von Quereinsteiger:innen oder der Aufbau eines Pflegeregisters zur Reaktivierung ehemaliger Pflegende sollten erprobt und systematisch weiterentwickelt werden.

Auf Makroebene sind klare gesetzliche Vorgaben, eine stärkere politische und gesellschaftliche Wertschätzung der Pflege, ihre Einbindung in Entscheidungsprozesse sowie eine evidenzbasierten Pflegeforschung erforderlich, die konkrete Empfehlungen zu Strukturen, Bedürfnissen und Arbeitsorganisation liefert. Leitlinien für den Krisenfall müssen trans- und interdisziplinär entwickelt werden und praktisch anwendbar sein sowie regelmäßig überprüft werden. Es bedarf zu dem einer stärkeren Berücksichtigung ethischer Fragen. Eine berufsständige Interessenvertretung könnte die Anliegen der Pflege systematisch in politische Entscheidungsprozesse einbringen. Außerdem ist eine klare Kompetenzzuweisung für die Pflege nötig, um Handlungssicherheit zu gewährleisten. Interprofessionelle Kooperation und der Dialog zwischen Praxis, Politik und Wissenschaft wurden als Grundpfeiler eines kohärenten Pandemiemanagements identifiziert.

Viele der vorgeschlagenen Maßnahmen auf der Mikroebene – etwa im Bereich der Arbeitsorganisation – bedingen strukturelle Anpassungen auf der Meso- und Makroebene. Dies betrifft unter anderem die Ressourcenverteilung (wie Schutzkleidung, Personal), gesetzliche Rahmenbedingungen und den Abbau ökonomischer Zwänge. Profitorientierte Strukturen wurden in beiden Workshops als hinderlich für resilientes Gesundheitssystem wahrgenommen.

Die Workshops führten zur Identifikation zahlreicher Maßnahmen und thematischer Schwerpunkte. Deren konkrete Ausgestaltung und Umsetzung sollten in zukünftigen Forschungsschritten oder im Abgleich mit vorhandener Evidenz weiter spezifiziert werden.

## Implikationen für die Forschung

Trotz des Fokus auf die (akut-)stationäre Pflege zeigte sich Forschungsbedarf auch in angrenzenden Bereichen wie ambulanter Pflege, Eingliederungshilfe und psychiatrischer Versorgung.

Zu den im Projekt entwickelten Maßnahmen bedarf es weiterer Forschung, insbesondere zur partizipativen Einbindung von Pflegenden im Pandemiemanagement, Netzwerkbildung, internen Kommunikation und Auswirkungen von New-Work-Ansätzen auf Teamstrukturen und Entscheidungsprozesse.

Ein weiterer Forschungsschwerpunkt sollte der psychischen Gesundheit von Pflegepersonen gelten – sowohl in der Krisenreaktion, der präventiven Alltagsgestaltung und der strukturierten Nachsorge. Formate wie Supervision, psychosoziale Begleitung und Resilienz fördernde Arbeitszeitmodelle bedürfen evidenzbasierter Bewertung im Kontext einer Krise und institutioneller Verankerung 5 21 .

Schließlich sollten Futures Literacy-Ansätze zur Förderung strategischer Zukunftskompetenz genutzt werden, um begrenzte Vorstellungsräume in Gesellschaft und im Gesundheitswesen zu überwinden.

## Schlussfolgerung

Die Ergebnisse der Szenario-Workshops verdeutlichen, dass effektives Krisenmanagement in stationären Pflegeeinrichtungen nur durch einen systemischen Ansatz realisiert werden kann, der Maßnahmen auf allen Ebenen – Mikro, Meso und Makro – integriert. Ein systemisches Zusammenspiel von Politik, Wissenschaft und Praxis ist unerlässlich, um die Krisenresilienz der Pflege zu stärken und die Arbeitsbedingungen nachhaltig zu verbessern. Dabei sollten vor allem die vier Themenfelder Kommunikation, interne Organisation, Schnittstellenmanagement und der Einbezug von Pflegepraktiker:innen berücksichtigt werden. Szenario-Workshops können dabei unterstützen (akut-)stationäre Einrichtungen auf Krisensituationen vorzubereiten und somit ein krisenfesteres Gesundheitssystem in Deutschland zu schaffen.

## Fazit für die Praxis

Abb. 3 informiert darüber, wie Aspekte der vier zentralen Themenfelder in der Praxis berücksichtigt werden könnten.

**Abb 3:**
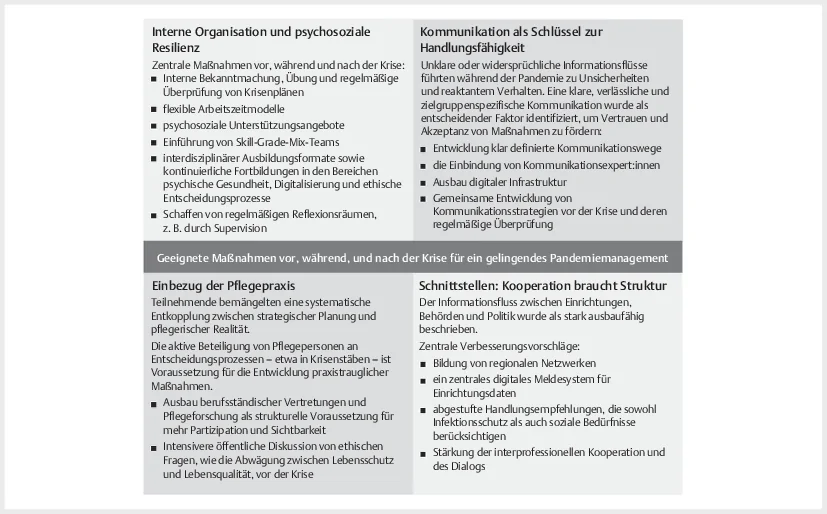
Empfehlungen für die Praxis.

## Fördermittel

Bundesanstalt für Arbeitsschutz und Arbeitsmedizin — http://dx.doi.org/10.13039/501100006522; F2541
